# The Systematic Review and Meta-Analysis on the Immunogenicity and Safety of the Tuberculosis Subunit Vaccines M72/AS01_E_ and MVA85A

**DOI:** 10.3389/fimmu.2020.01806

**Published:** 2020-10-08

**Authors:** Inayat Ullah, Shaheen Bibi, Ijaz Ul Haq, Safia, Kifayat Ullah, Long Ge, Xintong Shi, Ma Bin, Hongxia Niu, Jinhui Tian, Bingdong Zhu

**Affiliations:** ^1^Lanzhou Center for Tuberculosis Research and Institute of Pathogen Biology, School of Basic Medical Sciences, Lanzhou University, Lanzhou, China; ^2^Gansu Provincial Key Laboratory of Evidence Based Medicine and Clinical Translation, Lanzhou University, Lanzhou, China; ^3^School of Life Science, Northwest Normal University, Lanzhou, China; ^4^College of Chemistry and Chemical Engineering, Northwest Normal University, Lanzhou, China; ^5^Pakistan Institute of Community Ophthalmology (PICO), Hayatabad Medical Complex, KMU, Peshawar, Pakistan; ^6^Department of Biosciences, COMSATS University Islamabad, Islamabad, Pakistan; ^7^The First Clinical Medical College of Lanzhou University, Lanzhou, China

**Keywords:** subunit vaccine, tuberculosis, M72/AS01_E_, MVA85A, immunogenicity, safety, systematic review, meta-analysis

## Abstract

**Background:** Tuberculosis (TB) is a severe infectious disease with devastating effects on global public health. No TB vaccine has yet been approved for use on latent TB infections and healthy adults. In this study, we performed a systematic review and meta-analysis to evaluate the immunogenicity and safety of the M72/AS01_E_ and MVA85A subunit vaccines. The M72/AS01_E_ is a novel peptide-based vaccine currently in progress, which may increase the protection level against TB infection. The MVA85A was a viral vector-based TB subunit vaccine being used in the clinical trials. The vaccines mentioned above have been studied in various phase I/II clinical trials. Immunogenicity and safety is the first consideration for TB vaccine development.

**Methods:** The PubMed, Embase, and Cochrane Library databases were searched for published studies (until October 2019) to find out information on the M72/AS01_E_ and MVA85A candidate vaccines. The meta-analysis was conducted by applying the standard methods and processes established by the Cochrane Collaboration.

**Results:** Five eligible randomized clinical trials (RCTs) were selected for the meta-analysis of M72/AS01E candidate vaccines. The analysis revealed that the M72/AS01E subunit vaccine had an abundance of polyfunctional M72-specific CD4+ T cells [standardized mean difference (SMD) = 2.37] in the vaccine group versus the control group, the highest seropositivity rate [relative risk (RR) = 5.09]. The M72/AS01E vaccinated group were found to be at high risk of local injection site redness (RR = 2.64), headache (RR = 1.59), malaise (RR = 3.55), myalgia (RR = 2.27), fatigue (RR = 2.16), pain (RR = 3.99), swelling (RR = 5.09), and fever (RR = 2.04) compared to the control groups. The incidences of common adverse events of M72/AS01E were local injection site redness, headache, malaise, myalgia, fatigue, pain, swelling, fever, etc. Six eligible RCTs were selected for the meta-analysis on MVA85A candidate vaccines. The analysis revealed that the subunit vaccine MVA85A had a higher abundance of overall pooled proportion polyfunctional MVA85A-specific CD4+ T cells SMD = 2.41 in the vaccine group vs. the control group, with the highest seropositivity rate [estimation rate (ER) = 0.55]. The MVA85A vaccinated group were found to be at high risk of local injection site redness (ER = 0.55), headache (ER = 0.40), malaise (ER = 0.29), pain (ER = 0.54), myalgia (ER = 0.31), and fever (ER = 0.20). The incidences of common adverse events of MVA85A were local injection site redness, headache, malaise, pain, myalgia, fever, etc.

**Conclusion:** The M72/AS01_E_ and MVA85A vaccines against TB are safe and had immunogenicity in diverse clinical trials. The M72/AS01_E_ and MVA85A vaccines are associated with a mild adverse reaction. The meta-analysis on immunogenicity and safety of M72/AS01_E_ and MVA85A vaccines provides useful information for the evaluation of available subunit vaccines in the clinic.

## Introduction

Tuberculosis (TB) is a severe infectious disease with devastating effects on global public health. The World Health Organization (WHO) has estimated that one-third of the world population, ~2.2 billion individuals were latently infected with *Mycobacterium tuberculosis* (*M. tuberculosis*). The WHO Global TB report issued in 2017 showed that 10.0 million people had developed TB disease, which was found in men, women, and children in numbers of 5.8, 3.2, and 1.0 million, respectively (1). Today, latent TB infections and the progression of new diseases of *M. tuberculosis* in children are prevented by using the BCG vaccination. The merely approved BCG vaccine against TB has induced protective memory that continues for 10–20 years (1–3). However, BCG has not been capable of inhibiting pulmonary TB, the most common form of the disease, at any age of life cycle (4). In the current era, the prevalence of TB is high due to the appearance of multidrug-resistant TB, extremely drug-resistant TB, and human immunodeficiency virus (HIV)/TB co-infection. Therefore, there is, at present, a high demand for the construction of a safe and effective TB vaccine.

The M72/AS01_E_ candidate vaccine is a fusion protein, constructed from two *M. tuberculosis* immunogenic antigens Mtb39A and Mtb32A, combined with adjuvant system AS01_E_ (5). The Mtb39A (alternate gene name, Rv1196), which encodes a 39-kDa protein, a membrane-associated protein is an early expression in the life cycle of *M. tuberculosis* (6, 7). The Mtb39A antigen has been identified as an immune evasion factor present in the *M. tuberculosis* lysate. The purified recombinant Mtb39A stimulated strong T-cell proliferative and gamma interferon responses in peripheral blood mononuclear cells (PBMC) from nine of the 12 purified protein derivative (PPD)-positive individuals tested, and overlapping peptides were used to identify a minimum of 10 distinct T-cell epitopes. Furthermore, mice immunized with Mtb39A DNA have been shown to have increased protection from *M. tuberculosis*, indicated by a reduction of the bacterial load. The human T-cell responses and early animal studies provide support for further evaluation of this antigen as a possible component of a subunit vaccine for *M. tuberculosis* (8). The recombinant protein, Mtb32A was evaluated *in vitro* assays with donor PBMC from healthy PPD-positive individuals of diverse ethnic backgrounds. Mtb32A stimulated PBMC from healthy PPD-positive donors but not from PPD-negative donors to proliferate and secrete gamma interferon. The Mtb32A is secreted protein and the possible role of Mtb32 serine proteases as a virulence factor (s) during *Mycobacterium* spp. infection (9). A point mutation was made in the Mtb32A antigen to improve the long-term stability of M72 (10). The *two M. tuberculosis* antigens Mtb39A and Mtb32 were combined with the adjuvant system AS01_E_, containing monophosphoryl lipid A and *Quillaja Saponaria* Molina fraction 21, in a liposomal suspension, which was adjusted to induce a Th1 immune response (11). The clinical trials of the M72/AS01_E_ vaccine in adults and adolescents infected with *M. tuberculosis* had a clinically satisfactory profile and provoked great scale M72-specific humoral with CD4^+^ T-cell responses (12–18).

The MVA85A candidate vaccine was a viral vector-based vaccine, constructed from *mycobacterial* antigen 85A with delivery system MVA (Modified Vaccinia Ankara virus) to increase the protective efficacy of BCG (19). MVA85A has shown protection against *M. tuberculosis* in pre-clinical animal models (20). The high immunogenic results of the MVA85A vaccine by aerosol route in non-human primates are recommended for the evaluation of vaccination in clinical trials, particularly in humans (6). The first phase I clinical trials of MVA85A in healthy adults was reported in 2004 (19). The MVA85A vaccine was safety and immunogenicity assessed in various phase I/II clinical trials of patients that were HIV-positive (HIV^+^) or HIV-negative (HIV^−^) (21), healthy (22), *M. tuberculosis*-infected (23), and BCG vaccinated and non-vaccinated populations (21).

In this work, one protein/adjuvant-based subunit vaccine M72/AS01_E_, and one viral vector-based subunit vaccine MVA85A for meta-analysis were selected. The objective of the current analysis was to evaluate the immunogenicity and safety of M72/AS01_E_ and MVA85A in populations that were BCG vaccinated and non-vaccinated, HIV-positive, and negative, and even, in *M. tuberculosis*-infected populations. A literature review on their safety may provide an important reference to the proposed work and other TB vaccine candidates in the future.

## Materials and Methods

### Search Strategy

This systematic review was designed according to the preferred reporting items for systematic reviews and meta-analyses (PRISMA) guidelines (24). The PubMed, Embase, and Cochrane Library databases were searched extensively for published studies up until October 2019, to find out about M72/AS01_E_ and MVA85A candidate vaccines. Ethical approval was not required, as determined by the safety and immunogenicity of tuberculosis subunit vaccines: a systematic review and meta-analysis.

### Inclusion and Exclusion Criteria

The Inclusion and exclusion criteria were assessed via randomized clinical trials (RCTs) of the M72/AS01_E_ and MVA85A candidate TB vaccines, and a control group (e.g., placebo, adjuvant, or other vaccines). The inclusion criteria for the proposed studies opted for the evaluation of at least one result associated with the immunogenicity and safety of the vaccines in various populations and the intradermal treatment of two doses of M72/AS01_E_ and MVA85A or control. The HIV-infected or TB infected community were also included. The first result of interest was the serotype-specific M72/AS01_E_ and MVA85A antibody response, which had considered protective. The secondary outcome was the occurrence of adverse effects linked to the candidate vaccines. We excluded studies that did not report results of interest and those in which the data was unclear and/or duplicated in other reports.

### Study Selection

All related full text papers were collected sequentially, and the reference lists of every article were analyzed for single-arm studies.

### Data Extraction

Single-arm studies were included, as where the experimental arm of randomized controlled trials. For each review, the country of origin, year of publication, numbers of participants enrolled in TB-endemic areas, and other relevant information was recorded. All data were extracted according to the criteria for the systematic review of interventions outlined in the Cochrane handbook (25).

### Quality of Evidence and Risk of Bias

The risk of bias for each randomized clinical trial was estimated by applying a methodology recognized by the Cochrane collaboration (26). The Cochrane analysis stipulates that the results of an intervention should be based on the legality of the data collected from the included trials. This comprises a judgement and support of the judgement for each entry in a “risk of bias table,” in which each entry addresses a specific feature of the study. The judgement for each entry determines the risk of bias as “low risk,” “high risk,” or “unclear risk.” The last category indicates either lack of information or uncertainty over the potential for bias.

### Statistical Analysis

Microsoft Excel (Microsoft Corp. Albuquerque, NM, USA) was used for data collection, and included the randomized clinical trials. The Stata/SE (Stata Corp, College Station, TX, USA) software was used for the statistical analyses. Stata/SE was used for the meta-analysis and calculation of heterogeneity. The results were reported as relative risk (RR), estimation rate (ES), and standardized mean difference (SMD) with 95% confidence intervals (95%CI). The pooled proportion and 95% confidence interval (CI) were calculated for the adverse events of the M72/AS01_E_ and MVA85A vaccines. The statistical heterogeneity was tested among studies with the Q and *I*^2^-tests. A forest plot and funnel plot were generated to judge the overall effect size and determine the presence of publication bias. The *I*^2^ statistics, if the *p* ≥ 0.1 and *I*^2^ ≤ 50%, proposed that there was no statistical heterogeneity, and the fixed effects model was used for meta-analysis. Subsequently, if the *P* < 0.1 and *I*^2^ > 50%, it proposed that a random-effects model would be used, which could be explored through regression analysis. For trials including more than one treatment/control group, we used the data from the combined treatment/control groups. Publication bias was assessed using the RevMan 5.2 software and presented in the risk of bias summary diagram. Application of GraphPad Prism 6 was used to represent difference Statistics and draw figures among groups.

## Results

### Characteristics of the Included Studies

#### M72/AS01_E_

**Figure 1:**
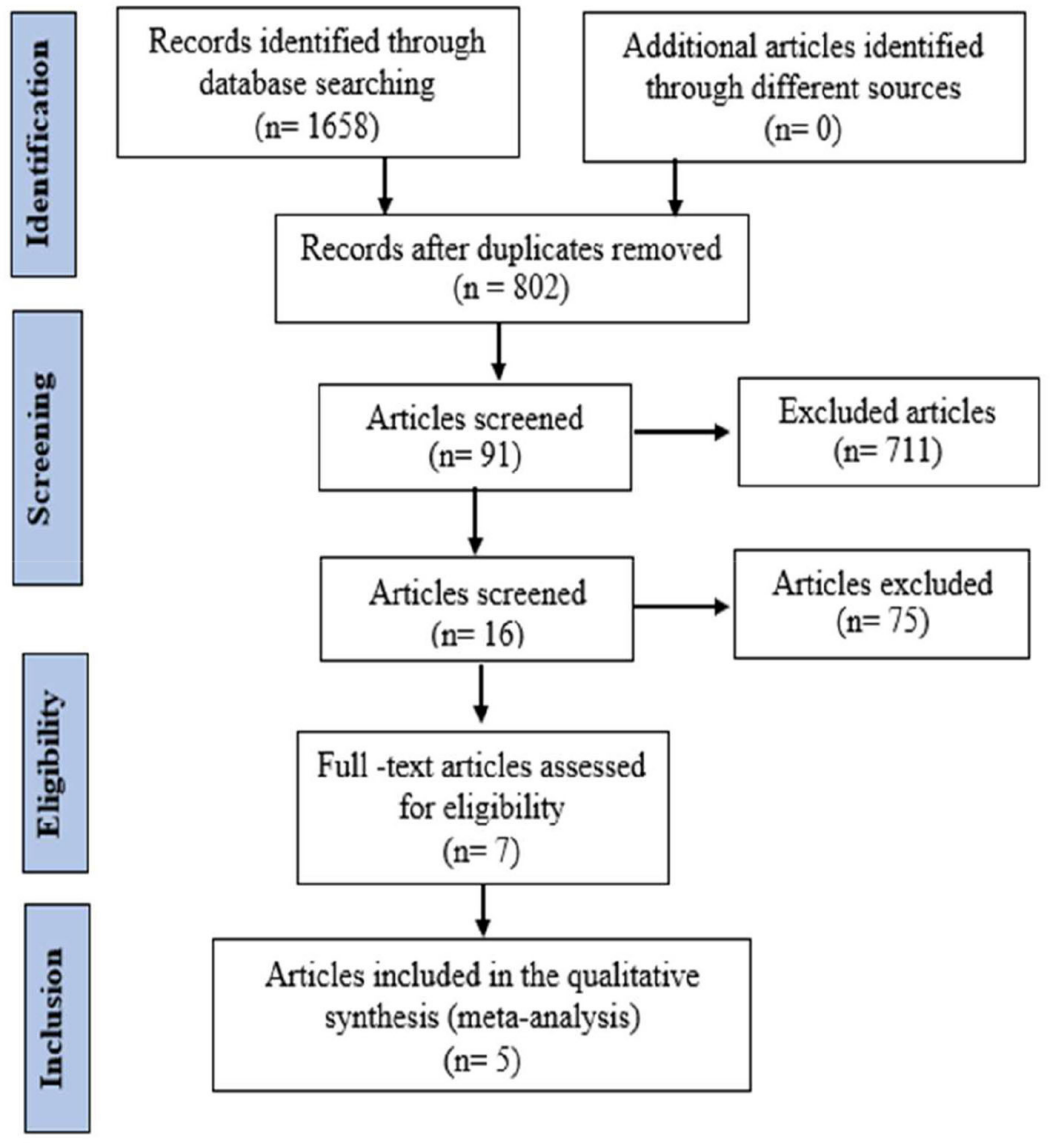
Study flow diagram of M72/AS01_E_.

**T**he findings for M72/AS01_E_ are shown in Figure 1, which shows an electronic search in which 1,658 records were identified, of which 802 records were included after duplicates were removed. After screening titles and abstracts, 91 full-text articles were considered, and seven for eligibility. Finally, five studies satisfied the standard eligibility criteria (double-blinded, one, two-arm RCTs) on M72/AS01_E_ (12, 13, 15, 18, 27). The key characteristics of the M72/AS01_E_ clinical trials included are described in Table 1.

**Table 1:** Characteristics of the M72/AS01_E_ studies included in the systematic review.

**References**	**Design**	**Country**	**Population**	**Age**	**Male/female**	**Followed-up**	**Year**	**Groups (*N*) and dosage 40 μg, 10 μg**
Montoya et al. (12)	Phase II RCT	Philippines	PPD-positive 3–10 mm	18–45 years	38/142	6 months	2013	M72/AS01_B_ (*N* = 40), M72/AS01_E_ (*N =* 40), M72/AS01_E_ (*N =* 40), M72/AS02_D_ (*N =* 40), M72/Saline (*N =* 10) AS01B, alone (*N =* 10)
Idoko et al. (15)	Phase II RCT	Gambia	BCG-vaccinated infants;	2–7 months	159/141	6 months	2014	Dose-outside EPI, 1 dose M72/AS01_E_ (*N =* 50), 2 doses M72/AS01_E_ (*N =* 50) Control (*N =* 50), Dose-within EPI, 1 dose M72/AS01_E_ (*N =* 52), 2 dosesM72/AS01_E_ (*N =* 49), EPI only (*N =* 49)
Penn-Nicholson et al. (13)	Phase II RCT	South Africa	HIV-negative adolescents;	13–17 years	31/29	6 months	2015	M72/AS01_E_ (*N =* 80), Saline (*N =* 38)
Gillard et al. (27)	Phase II RCT	Taiwan Estonia	Confirmed pulmonary TB; Treated pulmonary TB	18–59 years	82/60	6 months	2016	M72/AS01_E_ (*N =* 71) Saline (*N =* 71)
Van Der Meeren et al. (18)	Phase IIb RCT	KenyaSouth Africa Zambia	Healthy; Stable Chronic medical conditions	18–50 years	2,044/1,529	3 years	2018	M72/AS01_E_ (*N =* 1,786) Saline (*N =* 1,787)

*RCT, randomized controlled trial; PPD, tuberculin purified protein derivative; BCG, Bacillus Calmette–Guerin; HIV, human immunodeficiency virus; TB, tuberculosis; cART, combination anti-retroviral therapy*.

#### MVA85A

**Figure 2:**
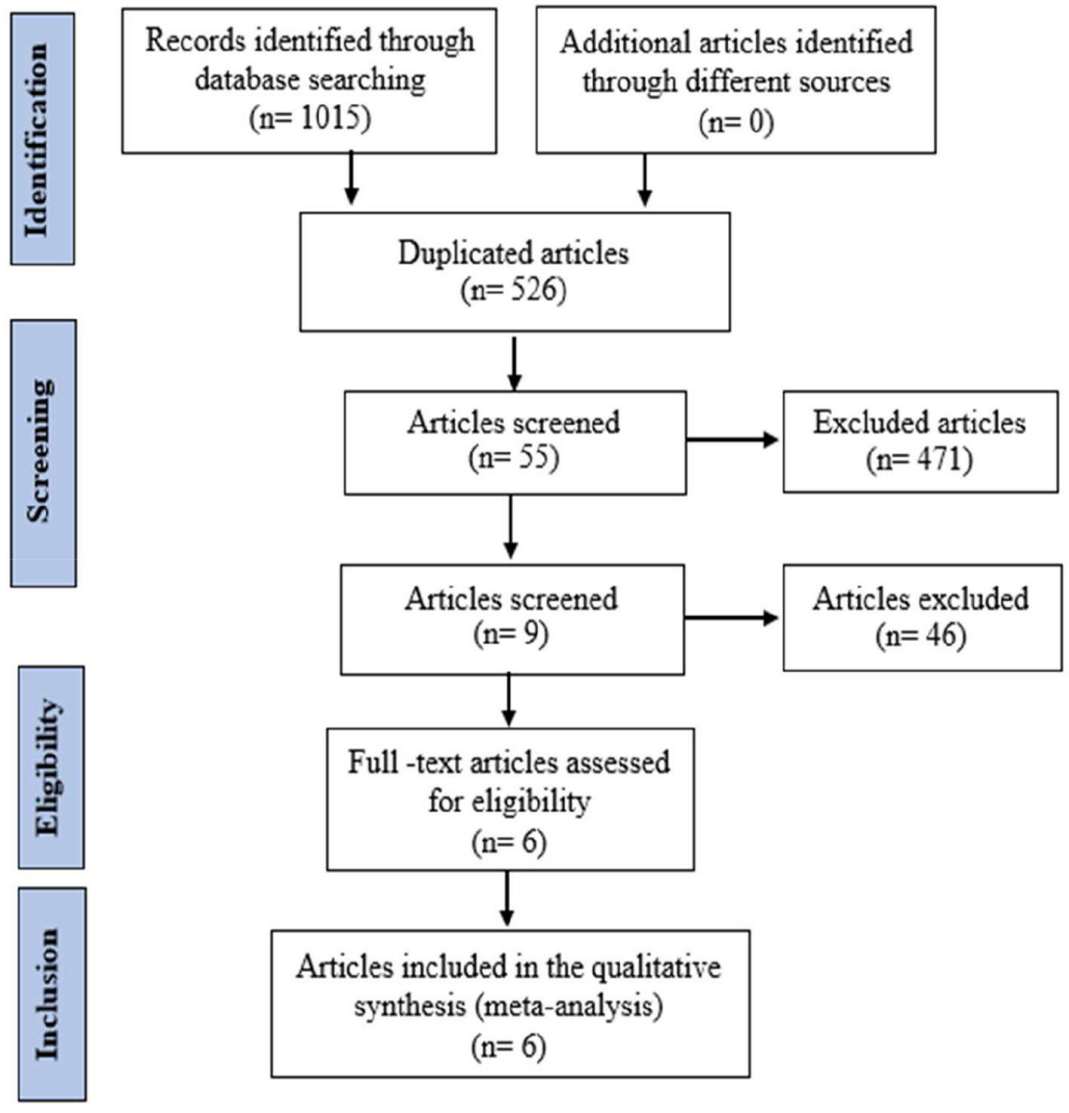
Study flow diagram of MVA85A.

Similarly, the findings for MVA85A are shown in Figure 2, which involved an electronic search in which 1,015 records were identified, of which 526 records were included after duplicates were removed. After screening the titles and abstracts, 55 full-text articles were considered, and nine for eligibility. Finally, six studies satisfied the standard eligibility criteria (double-blinded, one, two-arm RCTs) on MVA85A (23, 28–32) and were included in a meta-analysis. The key characteristics of the clinical trials for MVA85A are shown in Table 2.

**Table 2:** Characteristics of the MVA85A studies included in the systematic review.

**References**	**Design**	**Country**	**Population**	**Age**	**Male/female**	**Followed-up**	**Year**	**Dosage in pfu**
Pathan et al. (28)	Phase I	UK	healthy, HIV negative, BCG naive adults	18–55 years	17	6 month	2007	5 × 10^7^
Whelan et al. (29)	Phase I	UK	Healthy, HIV-negative, BCG vaccinated adults	22–54 years	10/4	6 month	2009	5 × 10^7^
Sander et al. (23)	Phase I	India, Africa, UK, Ireland	*M. tuberculosis* infected Individuals	20–49 years	10/2	6 month	2009	5 × 10^7^
Minassian et al. (30)	Phase I	Africa, Asia, Europe, America	HIV infected Individuals	21–52 years	9/1	6 month	2011	5 × 10^7^ and 10 × 10^7^
Pathan et al. (31)	Phase I	UK, Africa, Other	BCG-vaccinated volunteers	19–54 years	20 M	6 month	2012	5 × 10^7^ and 10 × 10^7^
Satti et al. (32)	Phase I	Europe, Africa	BCG-vaccinated volunteers	18–50 years	10/14	6 month	2014	10 × 10^7^

*Pfu, Plaque-forming units; BCG, Bacillus Calmette–Guerin; HIV, human immunodeficiency virus*.

Different routes rectified the M72/AS01_E_ and MVA85A subunit vaccine in diverse populations. The M72/AS01_E_ was administered intramuscularly, while the MVA85A was administered intradermally, except for a one-half trial of MVA85A, which was received by aerosol (32). In the low dose of MVA85A [5 × 10^7^ plaque-forming units (pfu)], the incidences of adverse events such as arthralgia, axillary lymph nodes (LN), fever, feverish, malaise, headache, myalgia, nausea, vomiting, and vasovagal syncope showed no significant heterogeneity because of the *I*^2^*-*value, which was reported as <50%. However, with the high dose of MVA85A (10 × 10^7^ pfu), the incidences of arthralgia, axillary LN, fever, feverish, malaise, headache, myalgia, nausea, vomiting, pain, pruritus, and redness showed statistically significant heterogeneity as their *P-*value was > 0.1. M72/AS01_E_ in both doses (40 and 10 μg) and induced incidences of adverse events such as chills, contusion, diarrhea, dizziness, dyspnea, eczema, feeling hot, glossitis, headache, hyperhidrosis, malaise, myalgia, nasopharyngitis, oropharyngeal pain, pain, productive, cough, pyrexia, and throat irritation. They showed no significant heterogeneity because of the *I*^2^*-*value, which was found to be <50%, but some adverse events like headache, pain, oropharyngeal pain, and nasopharyngitis showed statistically heterogeneity as their *P* value was more significant than 0.1.

### The Immunogenicity Evaluation of M72/AS01_E_

M72/AS01_E_ has induced potent M72-specific humoral and polyfunctional CD4^+^ T-cell mediated immune responses in adults treated for tuberculosis (27). M72/AS01_E_ was immunogenic in antiretroviral therapy (ART), stable and ART-naive, HIV-positive, and HIV-negative individuals. Regardless of their ART situation, this population of HIV positive subjects can mount cell-mediated and humoral responses to two M72/AS01_E_ doses, which persevere at 1-year post-vaccination. The M72/AS01_E_ vaccine at 7 days post-dose, induced polyfunctional M72-specific CD4^+^ T-cell responses (33). M72/AS01_E_ produced robust antibody and polyfunctional M72-specific CD4^+^ T cell responses remaining at 3 years, with the maximum CD4^+^ T cell responses detected in PPD negative adults (10). M72/AS01_E_ was a vaccine shown to be immunogenic in PPD-positive adults. M72/AS01_E_ induced anti-M72 humoral reactions and showed a long time polyfunctional M72-specific CD4^+^ T-cell response. IFN- γ was found in serum at 1 day post each vaccination (34). Clinically, the co-administration of M72/AS01_E_ with an expanded program of immunization (EPI) vaccines has no interference on their corresponding immunogenicity profiles. For the M72/AS01_E_ vaccine, two doses induced more immunogenicity than one dose (14).

### The M72/AS01_E_-specific CD4^+^ T-Cell

**Figure 3:**
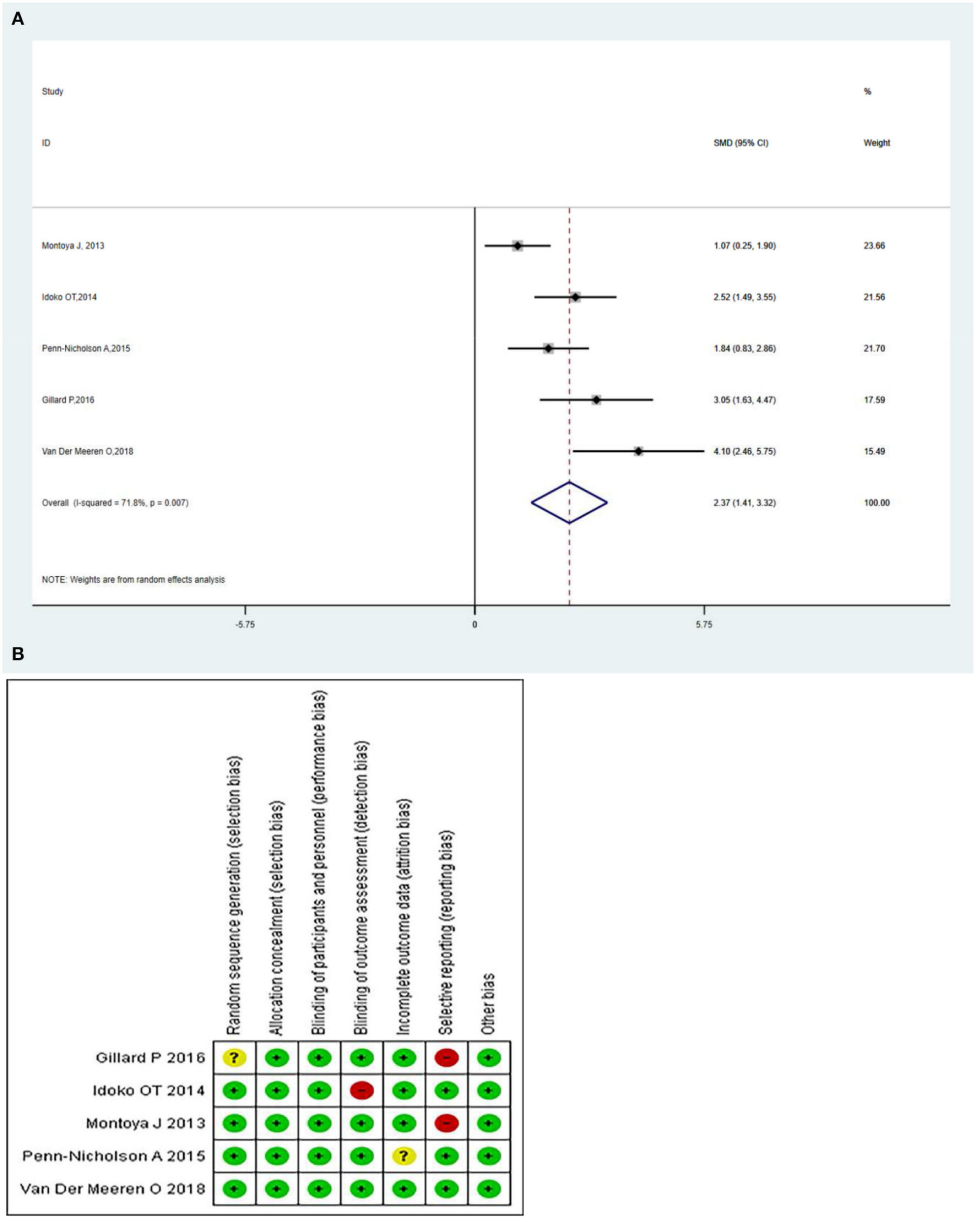
**(A)** Polyfunctional M72/AS01_E_-specific CD4^+^ T-cell evaluation. Forest plot: an SMD > 0 indicates that the vaccine can effectively stimulate the growth of polyfunctional CD4+ T-cells. SMD = 0, invalid result. Point estimates and 95%CI are shown for each study and the pooled results. A significantly higher abundance of polyfunctional M72-specific CD4^+^ T cells (SMD = 2.37) was observed in the vaccine group compared with the control group. **(B)** Methodological Quality and Risk of Bias summary of M72/AS01_E_.

The M72/AS01_E_-specific CD4^+^ T-cells produced more than two immune markers among cytokines IFN-γ, IL-2 TNF-α, IL-13, IL-17, and CD40L. The meta-analysis was conducted by analyzing the polyfunctional CD4^+^ T-cells of the vaccine compared with the control group. The overall mean value of CD4^+^ T-cells was changed using the natural logarithm (ln) form at different times. The results indicated a significant change between the vaccinated and non-vaccinated groups in the number of polyfunctional CD4^+^ T cells. As significant heterogeneity was reported (*I*^2^ > 50% and *P* < 0.1), a random-effects model was used. The overall pooled proportion of M72/AS01_E_-specific CD4^+^ T-cell was 2.37 (95%CI: 1.41, 3.32) (Figure 3A). The Methodological Quality and Risk of Bias summary of M72/AS01_E_, as seen in Figure 3B, showed no evidence of publication bias.

### The Safety Evaluation of M72/AS01_E_

**Figure 4:**
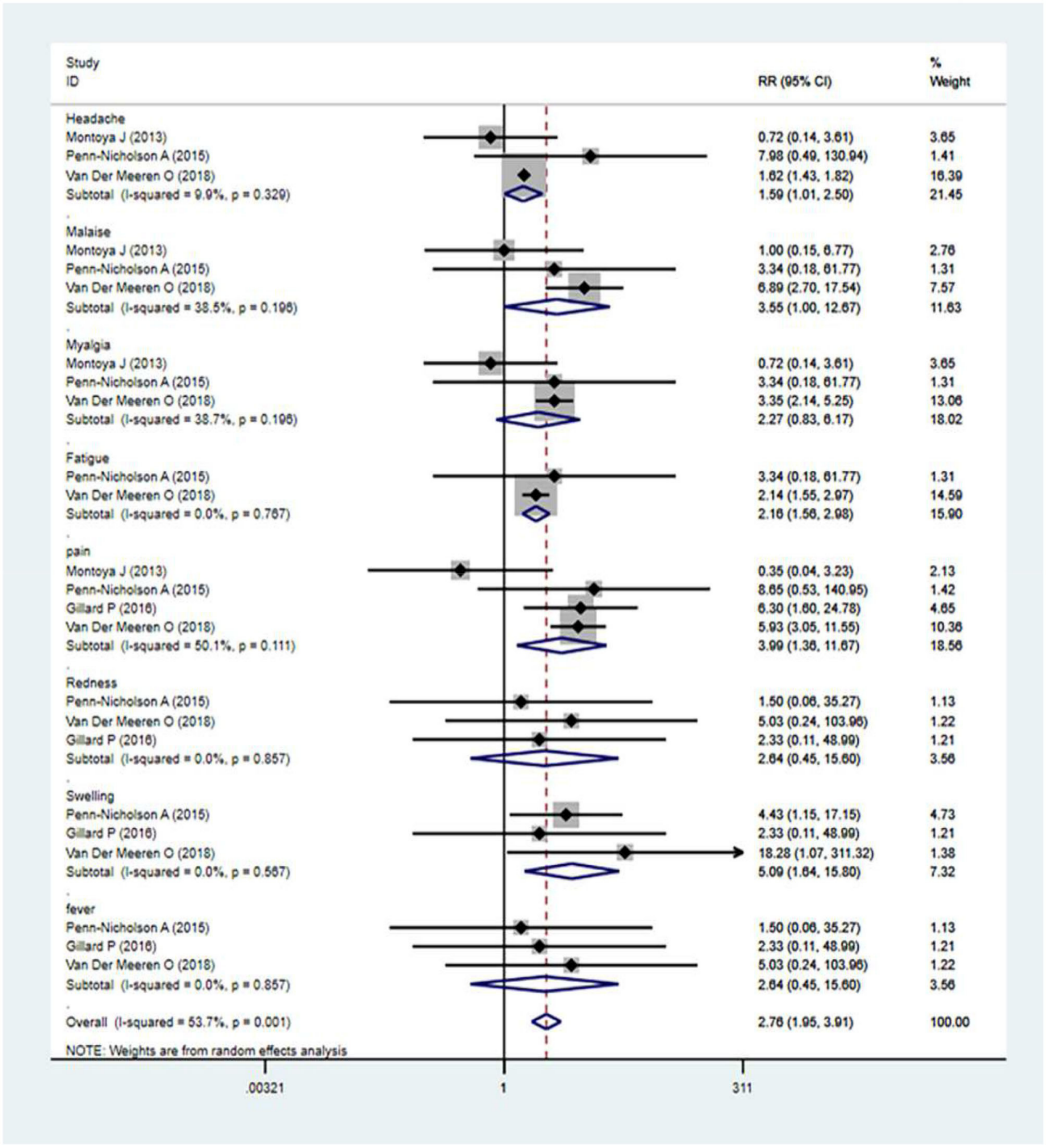
Safety evaluation of M72/AS01_E_. Forest plot: a RR >1 shows that the vaccine was protective; the result of the intersection with the intermediate invalid line was invalid. RR = 1, invalid result. Point estimates and 95%CI were presented for each clinical trial and the pooled results.

The local and systemic toxicity associated with the M72/AS01_E_ vaccine was assessed in five studies (12, 13, 15, 18, 27). Clinically, the M72/AS01_E_ vaccine had a tolerable safety profile when given to infants, either after or concurrently with EPI vaccines (14). Adverse events (AEs) usually occurred more in the vaccine group compared with the control. The incidences of common adverse events of M72/AS01_E_ were local injection site redness, headache, malaise, myalgia, fatigue, pain, swelling, fever, etc. The analysis revealed that the M72/AS01_E_ subunit vaccine's highest seropositivity adverse events rate was [relative risk (RR) = 5.09]. The M72/AS01_E_ vaccinated group were found to be at high risk of local injection site redness (RR = 2.64), headache (RR = 1.59), malaise (RR = 3.55), myalgia (RR = 2.27), fatigue (RR = 2.16), pain (RR = 3.99), swelling (RR = 5.09), and fever (RR = 2.04) as compared to the control groups (Figure 4).

### The Immunogenicity Evaluation of MVA85A

There was well-tolerated immunogenicity of the MVA85A vaccination in healthy adults, which induced a strong T cell response, as determined through the IFN-γ ELISPOT assay. The MVA85A-boosted BCG produced specific CD4^+^ T cells, which contained multiple populations of IL-2, IFN-γ, IL-17, and TNF-α as determined by polychromatic flow cytometry. The expression of IFN-γ, IL-2, TNF-α, and CD4^+^ T cells was increased throughout the peak BCG-specific response 7-days post-vaccination (35). MVA85A is highly immunogenic in individuals with latent TB infection (LTBI). Statistically, significant increases in Antigen 85A specific CD4^+^ T cells were founded after vaccination. An active antigen-specific IL-2 and IFN-γ response was induced by MVA85A, which was durable for 52 weeks (23). MVA85A did not significantly change either CD4 count or HIV RNA load during the evolution of the trial in either study group. The daily hematological and biochemical test results did not alter between study groups. The MVA85A vaccine was well-immunogenic in adults infected with HIV-1. The MVA85A vaccine induced a potent rise in antigen 85A-specific T-cell, which was mostly monofunctional and peaked 7 days after both vaccinations (36).

### The MVA85A-specific CD4^+^ T-Cell

**Figure 5:**
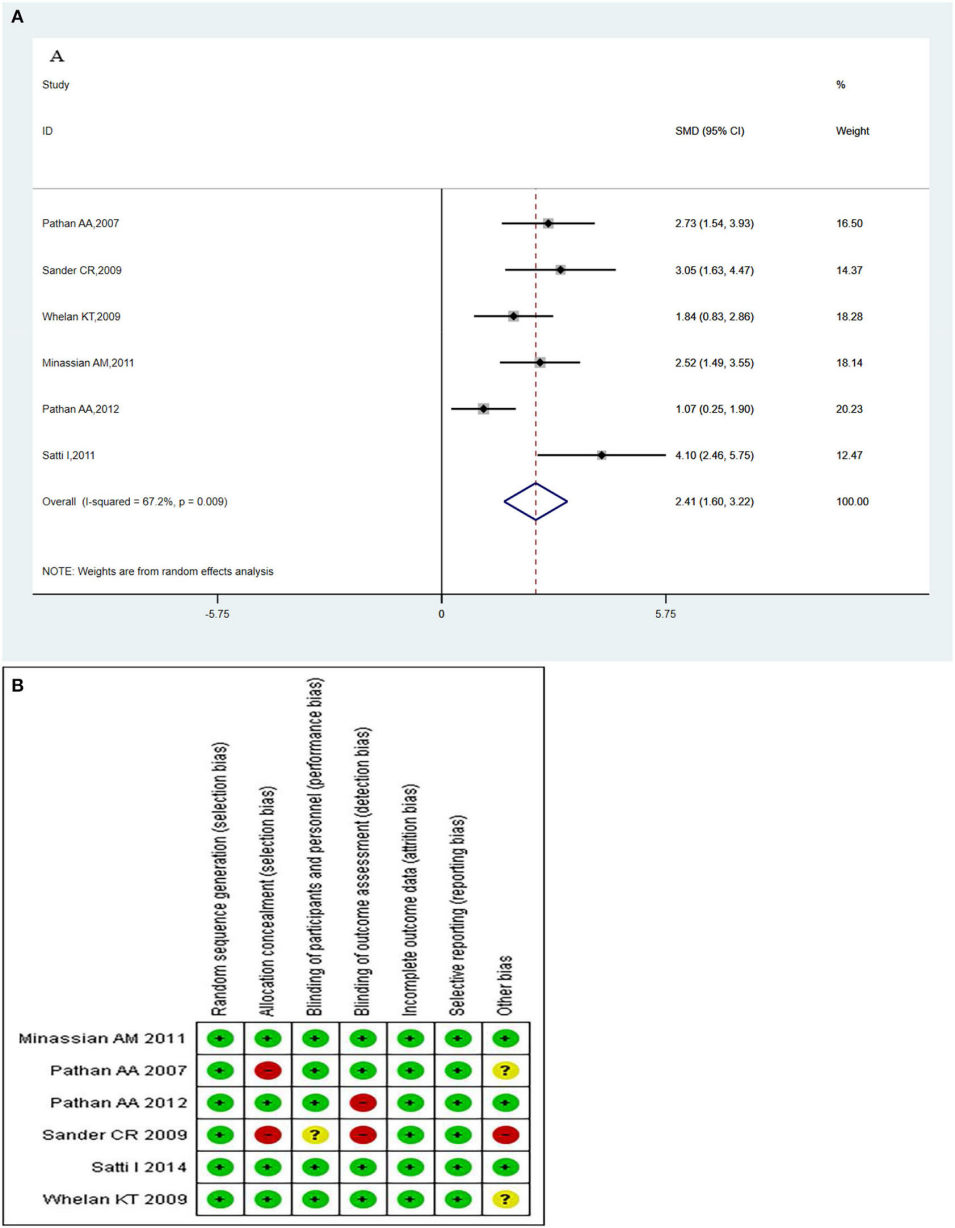
**(A)** Polyfunctional of MVA85A-specific CD4^+^ T-cell evaluation. Forest plot: an SMD > 0 indicates that vaccines can effectively stimulate the growth of polyfunctional CD4+ T-cells. SMD = 0, invalid result. Point estimates and 95%CI are shown for each study and the pooled results. Significantly higher abundantly of polyfunctional MVA85A-specific CD4^+^ T cells (SMD = 2.41) in the vaccine group compared with the control group. **(B)** Methodological quality and risk of bias summary of MVA85A.

The MVA85A -specific CD4^+^ T-cells produced more than two immune markers among cytokines IFN-γ, IL-2 TNF-α, IL-13, and IL-17. The meta-analysis was conducted by evaluating the polyfunctional CD4^+^ T-cells of the vaccine compared with the control group. The overall mean value of CD4^+^ T-cells was changed using the natural logarithm (ln) form at different times. The results indicated a significant change between the vaccinated and non-vaccinated groups in the number of polyfunctional CD4^+^ T cells. A random effects model was used because significant heterogeneity was reported (*I*^2^ > 50% and *P* < 0.1). The overall pooled proportion of MVA85A-specific CD4^+^ T-cell was 2.41 (95%CI: 1.60, 3.22) (Figure 5A). Methodological Quality and Risk of Bias summary of MVA85A shown in Figure 5B showed no evidence of publication bias.

### The Safety Evaluation of MVA85A

**Figure 6:**
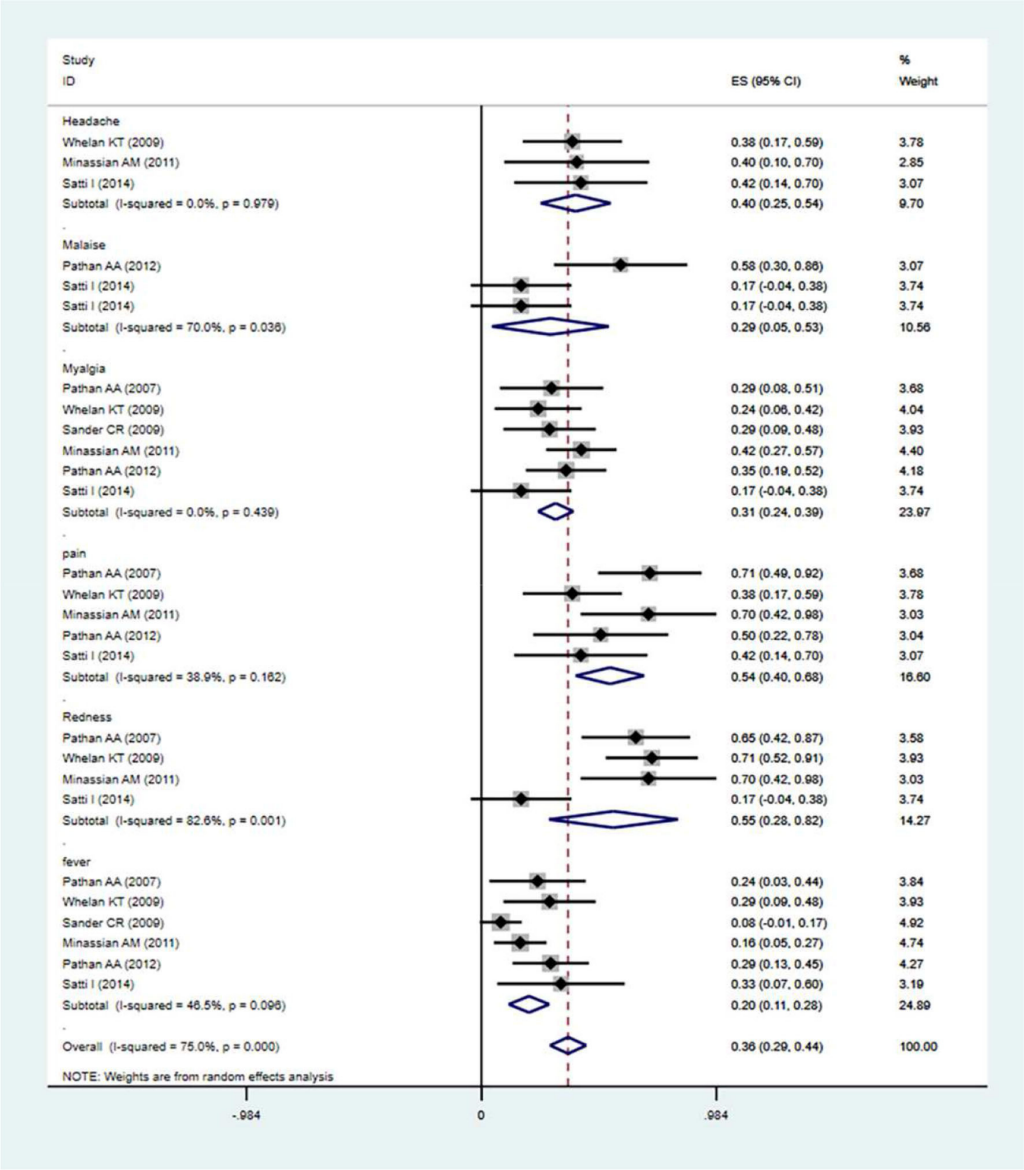
Safety evaluation of MVA85A. The estimation rate of headache, malaise, pain, redness, and fever, Point estimates, and 95%CI were described for each clinical trial and the pooled results.

The local and systemic toxicity associated with the MVA85A vaccine was assessed in five studies (23, 28–32). Generally, the profiles of the local adverse events described were not affected by the MVA85A doses that were tested, except for one report of severe swelling in the 1 × 10^7^ pfu group (31). The MVA85A vaccine-related to normal mild local intradermal injection-site reactions. Systemic adverse events did not considerably contrast between the two groups of aerosol MVA85A and intradermal saline placebo or intradermal MVA85A and aerosol saline placebo (32). Adverse events (AEs) occurred more usually in the vaccine group, compared with control. The analysis revealed that the MVA85A subunit vaccine's highest seropositivity adverse events rate was [estimation rate (ER) = 0.55]. The MVA85A vaccinated group were found to be at high risk of local injection site redness (ER = 0.55), headache (ER = 0.40), malaise (ER = 0.29), pain (ER = 0.54), myalgia (ER = 0.31), and fever (ER = 0.20). The main result from the random-effects meta-analysis is presented in Figure 6. Overall, the aggregated estimate across all six studies indicated adverse events of 0.36 (95% CI 0.29–0.44). The incidences of common adverse events of MVA85A were local injection site redness, headache, malaise, pain, myalgia, fever, etc.

## Discussion

The eradication of TB has been limited by the capability of *M. tuberculosis* to latently continue to be present in the human body without producing illness, a form stated as LTBI (37). It has been determined that nearly one-quarter of the global community has been infected by *M. tuberculosis*. Of those, 5–10% will change TB illness during their lifetime (1). Whereas, the majority of infected people are asymptomatic, they produce a robust acquired immune response to the pathogen (38). Therefore, the inhibition and therapy of LTBI is presently the locus of the ongoing investigation, and an extremely effective TB vaccine is needed to eliminate TB. This study was the first meta-analysis of clinical trials of TB subunit vaccines M72/AS01_E_ and MVA85A. The overall pooled results of estimated data in the case of MVA85A and M72/AS01_E_ showed that the two-subunit vaccines have general immunogenicity and clinical trials have indicated that they are safe.

Based on preclinical studies, protection against *M. tuberculosis* is arbitrated by antigen-specific polyfunctional CD4^+^ T cells (39–43). However, the immune associates of protection against TB have not been defined (44), IFN-γ, TNF-α, IL-2, and IL-17 are essential for the control of *mycobacterial* infection (45–47). IFN-γ and TNF-α can stimulate infected macrophages, respectively, which in chance prevent *M. tuberculosis* growth by inducing iNOS and autophagy (48, 49). Additionally, IFN-γ and TNF-α synergistically facilitate the killing of pathogens (49). IL-2 induces CD4^+^ and CD8^+^ T cell proliferation and differentiation and stimulates the growth of memory T cells during primary infection. IL-17 plays an essential antimicrobial pro-inflammatory part in the stages of *M. tuberculosis* infection by inducing neutrophil generation, stimulate cytokine production (50). Studies have shown that polyfunctional IFN-γ + IL-2+ TNF-α + CD4^+^ T cells may yield higher levels of each cytokine on a per-cell basis, compared with other T cells (43, 51).

M72/AS01_E_ vaccine was well-tolerated but had a higher frequency of slight to moderate local adverse events and severe pain at the injection site in the vaccinated compared to the placebo group. For M72/AS01_E_, pain, redness, headache, and myalgia were relatively common symptoms. Similarly, the MVA85A vaccine showed local injection-site reactions and other adverse events included mild influenza-like symptoms and local lymphadenopathy in most recipients. For MVA85A, the most common adverse events were induration, redness, pain, and headache. The profiles of reported local adverse events of M72/AS01_E_ were not affected by the doses tested. The three different doses of vaccines, M72/AS01_B_ (40 μg), M72/AS01_E_ (10 μg), and M72/AS01_E_ (20 μg), had comparable safety and reactogenicity profiles, which were similar to the result that developed in PPD-negative adults with M72/AS02_A_ and M72/AS01_B_ vaccines (both with 40 μg of M72). There were identical magnitudes and constancy in the stimulation of M72-specific CD4^+^ T-cell responses in the three M72 doses, and the two AS01 designs tested (10, 12).

The safety of the M72/AS01_E_ vaccine has completed several phases, which observed adults treated for TB disease, and adults with a history of treatment for TB disease. The study was terminated early because of an incidence of large injection site redness/swelling reactions in M72/AS01_E_ -vaccinated adults undergoing TB treatment. No other serious clinically related adverse events were observed (27). In clinical trials, the M72/AS01_E_ vaccine showed sufficient response of antigen-specific T-cells and antibody (52). Several types of preclinical studies have verified that humoral immunity may give protection against *M. tuberculosis* (53–55). M72/AS01_E_ vaccination-induced M72-specific antibodies persisted for a maximum of 3 years (56). In particular, two-doses of the vaccination seem to have strong long-term protection. Also, the AS01 adjuvant system is a part of the recombinant zoster vaccine (57) and RTS, S/AS01 malaria vaccine (58–60) (both recently studied in phase III studies). Adaptive immune responses (humoral and cellular) are linked to enhancement by AS01E. Therefore, AS01E may stimulate increases in Ag-specific levels of costimulatory molecules, cytokine release, and antibody responses in humans (61). The use of adjuvants is essential to induce the utmost strong immune responses. Hence, the use of a potent adjuvant such as AS01E may permit the decrease of antigen doses (i.e., antigen sparing effect). The M72/AS01_E_ subunit is the best choice in clinical practice.

The phase I clinical trial in HIV-infected adults in Senegal showed that MVA85A was well-tolerated and immunogenic, consistent with results from a UK clinical trial in HIV-infected subjects (30). The safety and immunogenicity profiles of MVA85A reported in a phase II trial with HIV-1 positive patients were similar to those in a HIV-1 negative trial (21, 23, 30, 35). The phase II trial, in healthy infants previously vaccinated with BCG, showed that MVA85A was safe and well-tolerated (22, 62). Both BCG-BCG and BCG-MVA85A immunization were well-tolerated with no severe vaccine-related local and systemic adverse events. It is necessary to point out that there is no significant protective efficacy against *M. tuberculosis* infection observed in infants when MVA85A was used to boost BCG-primed immunity. This lack of efficacy was not consistent with results from studies in animals, which proposed the potential for efficacy (6, 7).

### Strengths and Limitations

Our study had several strengths. First, this meta-analysis was the first systematic review and meta-analysis to evaluate the immunogenicity and safety of tuberculosis subunit vaccines M72/AS01_E_ and MVA85A. Second, this meta-analysis was based on up-to-date literature and has presented the largest scale synthesis to date of double-blinded, one, two-arm RCTs with large sample sizes, which increased the statistical power to detect potential associations. The vaccines mentioned above have been studied in various phase I/II clinical trials. A review of their immunogenicity and safety may give an essential reference for the work on other TB vaccine candidates in the future.

Our study has some limitations. A considerable degree of heterogeneity was still observed among the included trials. This might be due to the differences in populations, and different vaccine-administered routes for the two vaccines M72/AS01_E_ and MVA85A. The M72/AS01_E_ was administered intramuscularly, while MVA85A was administered intradermally, except for one half-trial of MVA85A, which was received by aerosol. There may also be other unknown biases in the studies examined.

## Conclusions

The findings of this meta-analysis study suggest that M72/AS01_E_ and MVA85A have immunogenicity and were generally found to be safe in populations that were BCG vaccinated and non-vaccinated, and in HIV-positive and negative, and even among populations who had previously been *M. tuberculosis-infected*. The meta-analysis on the immunogenicity and safety of the M72/AS01_E_, MVA85A vaccines provide some useful information for the evaluation of other subunit vaccines.

## Author Contributions

InU, BZ, and JT planned and designed the research. LG, JT, and InU provided methodological support/advice. InU, LG, SS, SB, and HN tested the feasibility of the study. InU, SB, SS, IjU, KU, and XS extract data. InU, LG, and IjU performed the statistical analysis. InU wrote the manuscript. All authors approved the final version of the manuscript.

## Conflict of Interest

The authors declare that the research was conducted in the absence of any commercial or financial relationships that could be construed as a potential conflict of interest.
